# Supplementary material for: Learning curve and surgical outcome of robotic assisted colorectal surgery with ERAS program
Source: Sci Rep. 2022 Nov 29;12:20566. doi: 10.1038/s41598-022-24665-w (PMC9709162; doi:10.1038/s41598-022-24665-w)
Supplement: Supplementary file 1 — Supplementary Information 1. [file 41598_2022_24665_MOESM1_ESM.zip › LSC Raw data-Ñ[▒K/062/N_ñw╝╨░O▒Kñσ.pdf]

W55 / W55

臺中榮民總醫院

[redacted]

## 基本資料

- 室別： 二醫手術室-31室，手術科別： CRS，手術類別： 常規，日期： 2020/01/15
- 進入時間： 1010 ； 麻醉開始時間： 1015 ； Time Out時間： 1055 ； 劃刀時間： 1055 ； 手術結束時間： 1525 送出時間： 1535 ；
- 麻醉方式： GE
  診斷：A colon cancer.
  手術名稱：Laparoscopic right colectomy and anastomosis
  手術局部用藥：無
  術前預防性抗生素：
  - 抗生素名稱一： Veterin 2GM IVD/由麻醉護理執行
  - 時間： 1055
  - 上次簽章: 彭馨慧

  ，追加預防性抗生素：
- 手術醫師： 林俊余，楊曙榮，陳威遠
- 麻醉醫師： VS 施涵薰
- 
  - 刷手護士A ： 林昱君，時間： 1010-1200，接替刷手一： 楊智雯，時間： 1200-1300，接替刷手二： 彭馨慧，時間： 1300-1535
  - 流動護士A ： 彭馨慧，時間： 1010-1230，接替流動一： 林昱君，時間： 1230，接替流動二： 張仟品，時間： 1500-1535

---

## 皮膚狀況

A-V Shunt： 無
術前： 完整
　記錄者： 上次簽章: 彭馨慧
術後： 完整
　記錄者： 張仟品

---

## 手術準備

手術臥式 單一手術臥式

- 
  - 手術臥式： 仰臥
  - 使用附件： 溫毯 38 ℃，頭圈
  - 皮膚準備： 10%Aq Betadine，Hibiscrub，其它： 2%Chlorhexcidine
  - 電燒： 使用電燒: 電極片位置： 其它： 床上迴路電極墊，記錄者： 上次簽章: 彭馨慧

止血帶T： 無

---

## 植入物

- 植入物名稱/號碼/數量/部位：Ethicon ligaclip ER420/colon/20釘
  Ethicon endo surgery 75mm x2 /colon

，記錄者： 上次簽章: 林昱君

---

## 標本送檢

- 細菌培養 無
- 細胞學 無
- 冰凍切片 無
- 病理標本
  組織名稱一：A-COLON
  ,
  組織名稱二：meddle colic root
- 截肢 無

標本交付 標本： 2 袋，標本： 0 盒，標本： 0 瓶，細胞學： 0 管，Culture Tube： 0 管，被交付醫師： CR 楊曙

榮，

- 記錄者： 上次簽章: 林昱君
- 時間： 1430

## 傷口術後數料

傷口數料： 傷口數料： 無

- 請於圖上標示部位：

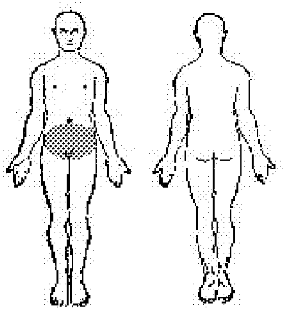

腹部五切口

- 傷口數料： menpor覆蓋

## 導管

| 導管 | 天數 | 導管資訊 | 插入日期 | 插入時間 | 更換日期 | 拔除日期 | 拔除時間 | 拔除原因 | 預計拔除/更換 | 預計拔除/更換日期 |
|---|---|---|---|---|---|---|---|---|---|---|
| Foley(1) | 1 | 號碼:16 Fr.; 部位:尿道; | 2020/01/15 | 1020 | | | | | | |
| Penrose Drain(1) | 1 | 號碼:12 mm.; 部位:肝下; | 2020/01/15 | 1430 | | | | | | |
| Sump NG Tube(1) | 1 | 號碼:16 Fr.; 部位:胃; | 2020/01/15 | 1040 | | | | | | |
| A-line (1) | 1 | 號碼:20 號; 部位:右手; | 2020/01/15 | 1025 | | | | | | |

## 用物計數

- 手術中出血量： 少 c.c.
- 手術中尿量： NIL c.c.
-
    - 其它： 無

用物計數： 多切口同時縫合

- 1.器械： 正確，2.縫針： 正確，3.刀片： 正確，4.紗布： 正確，5.腹部墊： 未使用，6.紗球： 未使用，7.PEANUTS ： 未使用，8.紗條： 未使用，9.絲帶： 未使用，10.針頭： 未使用，11.Vessel loop： 未使用，12.Boots： 未使用，13.Jelco： 未使用，14.棉條： 未使用，
    - 第一次：
        - 刷手護士 ： 彭馨慧
        - 流動護士： 上次簽章: 林昱君
        - 時間： 1420

    ，
    - 第二次：
        - 刷手護士 ： 彭馨慧
        - 流動護士： 上次簽章: 林昱君
        - 時間： 1430

    ，
    - 第三次：
        - 刷手護士 ： 彭馨慧
        - 流動護士： 上次簽章: 林昱君
        - 時間： 1440

## 備註

備註： NIL

- 記錄者： 上次簽章: 林昱君
- 時間： 1430

填寫護理人員 張仟品

正式摘要

W55 / W55

doctorRank:B
